# Can Large Language Models Simulate Spoken Human Conversations?

**DOI:** 10.1111/cogs.70106

**Published:** 2025-09-01

**Authors:** Eric Mayor, Lucas M. Bietti, Adrian Bangerter

**Affiliations:** ^1^ Department of Psychology University of Basel; ^2^ Department of Psychology Norwegian University of Science and Technology; ^3^ Institute of Work and Organizational Psychology University of Neuchâtel

**Keywords:** Spoken conversation, LLM to LLM conversation, Linguistic alignment, Conversational coordination, Computational methods

## Abstract

Large language models (LLMs) can emulate many aspects of human cognition and have been heralded as a potential paradigm shift. They are proficient in chat‐based conversation, but little is known about their ability to simulate spoken conversation. We investigated whether LLMs can simulate spoken human conversation. In Study 1, we compared transcripts of human telephone conversations from the Switchboard (SB) corpus to six corpora of transcripts generated by two powerful LLMs, GPT‐4 and Claude Sonnet 3.5, and two open‐source LLMs, Vicuna and Wayfarer, using different prompts designed to mimic SB participants’ instructions. We compared LLM and SB conversations in terms of alignment (conceptual, syntactic, and lexical), coordination markers, and coordination of openings and closings. We also documented qualitative features by which LLM conversations differ from SB conversations. In Study 2, we assessed whether humans can distinguish transcripts produced by LLMs from those of SB conversations. LLM conversations exhibited exaggerated alignment (and an increase in alignment as conversation unfolded) relative to human conversations, different and often inappropriate use of coordination markers, and were dissimilar to human conversations in openings and closings. LLM conversations did not consistently pass for SB conversations. Spoken conversations generated by LLMs are both qualitatively and quantitatively different from those of humans. This issue may evolve with better LLMs and more training on spoken conversation, but may also result from key differences between spoken conversation and chat.

## 1 Introduction

The potential use of large language models (LLMs) to simulate human cognition and behavior has been heralded as an upcoming paradigm shift in psychological and social science research (Grossmann et al., 2023; Ke et al., 2024; Pickering & Garrod, 2021; Salah et al., 2023). LLMs are capable of analogical reasoning to an extent that sometimes exceeds human performance (Webb et al., 2023). In silico samples created using GPT‐3 accurately emulate the ideas and attitudes of human groups (Argyle et al., 2023). GPT‐3 makes decisions comparable to humans’ and outperforms them in the multiarmed bandit task, but is mediocre at causal reasoning (Binz & Schulz, 2023). GPT‐3.5 and GPT‐4 can potentially solve false‐belief tasks (Kosinski, 2024), and moral judgment is similar in human participants and GPT3.5 (Dillion et al., 2023). However, LLMs may generate less diverse responses than human samples (Belem et al., 2024). For example, they may be subject to a “correct answer effect,” inappropriately treating opinion questions as if there was a correct answer and producing near‐zero variability in responses (Park et al., 2024).

LLMs may also be useful for simulating collective behavior (Park et al., 2023), especially as such behavior is mediated by language. For example, ChatGPT is proficient in many aspects of human language use, including summarization, text classification, translation, and question‐answering (e.g., Wei et al., 2022), and can interpret word meanings in context and assess sentence grammaticality. It can make human‐like grammaticality judgments (Qiu et al., 2024), adapt lexical choice to the identity of the interlocutor (Cai et al., 2023), and reuse syntactic structure, for example, Michaelov et al. (2023) (see Cai et al., 2023 for further experiments comparing LLM and human language use). Accordingly, LLMs could potentially “simulate various social interactions, from informal exchanges to complex dialogues” (Salah et al., 2023, p. 2). Using dialogue generated by LLMs in research instead of humans is an attractive prospect for different reasons, for example, freeing research teams from participant recruitment and speech transcription, or affording better experimental control. For example, synthetic telephone call conversations might be useful for improving automatic segmentation and tagging of human telephone calls (Malkiel et al., 2023), provided that synthetic and human dialogical productions are similar.

Emerging research comparing LLM dialogue production to humans shows mixed results. On the one hand, LLMs can arguably pass a Turing test (Jones & Bergen, 2023, 2024), on the other hand, comparisons of their simulated dialogue in a human‐LLM conversation reveal their limitations. In a study using the WildChat corpus (Zhao et al., 2024), LLMs were not able to replicate human responses. Performance was better for semantic metrics, but worse for syntax and style. Notably, LLMs had trouble simulating humans’ tendencies to end conversations, potentially highlighting a deficit in models’ understanding of conversational intentions (Ivey et al., 2024).

Most studies investigate the abilities of LLMs to simulate human conversations in a chat, that is, *written*, environment. The interactive, open‐ended nature of human dialogue may be more difficult to emulate by LLMs in *spoken* conversations in a real‐time medium (e.g., face‐to‐face, telephone, or video‐mediated). Language production and comprehension differ fundamentally between these media (Umair et al., 2024). Because of the evanescence of speech (Clark & Brennan, 1991), participants in spoken conversations adapt to each other's moves on the fly, preparing their response while decoding a partner's talk (Levinson, 2016). This creates tighter coordination demands, and spoken conversations feature devices to coordinate conversational moves and understanding of each other's intentions (Bangerter & Clark, 2003; Dideriksen et al., 2023; Holler & Levinson, 2019; Schiffrin, 1987). These may include discourse markers, like *oh*, which signals a change in the speaker's mental state (Heritage, 1984). Another ubiquitous device in conversation is constituted by back‐channel utterances (*uh‐huh*) that signal understanding (Dingemanse et al., 2022). Interactive procedures like repair are necessary to correct misunderstandings and other coordination failures (Dingemanse & Enfield, 2023). Coordination is also facilitated by the increasing *alignment* of participants’ contributions over the course of a conversation (Pickering & Garrod, 2006; Rasenberg et al., 2020; Riordan et al., 2014; Dideriksen et al., 2023). Alignment operates at lexical, syntactic, and conceptual levels (Duran et al., 2019). Lexical alignment involves repeating lexical items, and syntactic alignment repeating syntactic constructions across interlocutors’ utterances. Conceptual alignment is convergence on meaning (Duran et al., 2019; Garrod & Anderson, 1987), which might be in part compositionally emergent from lexical and syntactic alignment (Garrod & Anderson, 1987; Gandolfi et al., 2023; Pickering & Garrod, 2004; Bangerter et al., 2020). LLMs might be particularly prone to align contributions to conversations because of their documented sycophantic tendencies with humans, including exaggerated emotional validation, endorsing user actions, or refraining from challenging users (Cheng et al., 2025).

Further, human spoken conversations do not emerge out of a vacuum. Participants need to coordinate on the possibility of interacting together to even start conversing. This is accomplished in openings, where participants mutually identify themselves to each other, check availabilities, exchange greetings, or inquire about the well‐being of their partner (Pillet‐Shore, 2018). Depending on whether the conversation has been previously arranged (e.g., an appointment) or not (e.g., running into a friend on the street), coordinating on topic introduction may be necessary. Participants also use procedures to coordinate on shutting down, or closing, the conversation. This initially involves agreeing that both parties are ready to end the conversation. Because this is potentially face‐threatening, participants may accomplish this process implicitly, for example, by exchanging *okay*s (Schegloff & Sacks, 1973). If participants explicitly invoke ending the conversation, they tend to appeal to external reasons (Albert & Kessler, 1976). This may be followed by expressions of pleasure, summaries, continuity statements, and well‐wishing (Albert & Kessler, 1978).

Spoken conversational phenomena (repair, alignment, back‐channels, discourse markers, openings, and closings) are generated by a suite of special abilities and motivations human participants bring to social interactions, the human “interaction engine” (Levinson, 2020). These include a capacity for inferring intentions (e.g., theory of mind) and other mental states of coparticipants from their often ambiguous conversational productions as well as a sense of joint commitment, the mutual understanding of the rules, rights, and obligations that participants build up during the conversation (Clark, 2020; Bangerter et al., 2022). There is currently no evidence that LLMs experience joint commitment, and the evidence that they possess a theory of mind or can detect intentions is mixed (Belem et al., 2024; Ivey et al., 2024; Kosinski, 2024; Shapira et al., 2024). The question arises, then, whether they are able to simulate human spoken conversational phenomena. One might surmise that LLMs would excel at all things linguistic because of their construction. However, their pretraining data may often massively rely on written dialogue, and probably an abundance of fictional dialogue or protocols. In such texts, the spoken conversational phenomena described above are vastly underrepresented (Dingemanse & Liesenfeld, 2022). Thus, it is unclear whether LLMs are currently capable of generating realistic spoken conversational data with sufficient granularity for supporting scientific analyses and sufficient variability to capture human diversity. Indeed, recent studies suggest that even LLMs fine‐tuned on conversation specifically for investigating this question fail to replicate human phenomena like taking into account speaker identity in comprehension (Umair et al., 2024) or predicting potential transition relevance places for turn‐taking (Umair et al., 2024).

Beyond this question regarding the current abilities of LLMs, the case of spoken conversations is of theoretical interest. Spoken conversation is embodied in the physical world, in the case of telephone conversations, like in the Switchboard (SB) corpus, in real‐time comprehension, and production of audible linguistic signals using resource‐limited processes (Holler & Levinson, 2019). Spoken conversations are further grounded in a complex and ever‐changing agency, the expression and interpretation of which is interwoven with communicative signals described above (Bangerter et al., 2022). LLMs are neither embodied in the physical world (Torres‐Martínez, 2024), nor imbued with agency in the human sense (Barandiaran & Almendros, 2024). In some domains (sensory and motor), LLMs’ lack of embodied experience limits their ability to produce humanlike conceptual representations (Xu et al., 2023). Of course, it is possible that, in the future, with sufficient spoken conversational training data, LLMs may be able to approximate human spoken conversations. However, even if more appropriate training sets are used in the future and sufficient data is available (Dingemanse & Liesenfeld, 2022), a lack of agency and embodied experience may impede humanlike performance. For this reason, spoken conversation is a particularly relevant test case of gauging the limits of LLMs.

We conducted two studies to assess the capacities of LLMs to realistically simulate spoken conversational phenomena. In Study 1, we assessed the ability of LLMs to replicate objective, highly granular features of human spoken conversation. We prompted two powerful LLMs, GPT‐4 and Claude Sonnet 3.5, as well as two smaller fine‐tuned models, Vicuna 13B v1.5 16k (fine‐tuned from Llama 2 13B with chat conversations) and Wayfarer (fine‐tuned from Llama 3.3 70B with adversarial role‐play conversations), to generate human‐like telephone conversations, using the SB corpus (Godfrey et al., 1992) as a model. The SB corpus comprises around 2500 telephone conversations where participants were matched with strangers along demographic and regional criteria and instructed to talk about a specific topic. Some of these conversations are transcribed, including the opening and closing phases. We prompted LLMs to generate conversations as similar to SB as possible. Each LLM conversation was matched to the topic and to the demographic characteristics of participants of a corresponding SB conversation. We compared the LLM and SB conversations in terms of lexical, syntactic, and conceptual alignment (Duran et al., 2019), investigating whether SB and LLM conversations exhibit similar levels of alignment and whether they increase in alignment between *Earlier* and *Later* sections of the conversation.

We also compared the frequency of different speech particles used to coordinate conversation (we hereafter refer to these as *coordination markers*). We first investigated the discourse marker *oh*. Because *oh* typically signals a change of the mental state of the speaker (Fox Tree & Schrock, 1999), and because LLMs do not have mental states that can change, it is an open question whether they use *oh* appropriately. We also compared the frequency of the back‐channel *uh‐huh* in both corpora, because it is a potentially universal (Dingemanse et al., 2022) signal of positive understanding in listeners and thus an important conversational coordination device. Further, we compared the frequency of *okay* in both corpora. *Okay* is a multifunctional word used in many languages (Betz et al., 2021) to signal shifts, or transitions, between different parts of a joint action (Bangerter & Clark, 2003; Bangerter et al., 2024), for example, between opening phases and the main body of a conversation, or between the main body and the closing phase (Bangerter et al., 2004). Coordination markers like *oh*, *uh‐huh* and *okay* reflect human participants’ efforts to coordinate mental states in conversation. Finally, we also compared opening and closing phases in LLM and SB conversations, counting how frequent various features (e.g., introductions for openings, well‐wishing for closings) were in each corpus.

We tested different LLMs using different combinations of prompts and turn‐generation procedures (Table 1).^1^ In a *basic prompt*, we instructed LLMs to generate phone conversation dialogue about a certain topic (matching SB conversations), which would last for 50 turns of talk, and thus not to initiate the closing too early, with roles matching the corresponding SB conversations. After initial results showed that GPT4‐1 produced reduced opening and closing features, we also used an *opening and closing* prompt, which additionally instructed LLMs to open the conversation by greeting partners and producing small talk (e.g., introductions), and to close the conversation politely (e.g., expressing pleasure, continuity, or well‐wishing). For similar reasons, after initial analyses of GPT4‐1, we also used a *coordination marker* prompt that additionally instructed LLMs to employ coordination markers to sound natural. Finally, we also used a variation prompt for one corpus (Claude‐2), which instructed LLMs not to use the same response format over successive turns.

**Table 1:** LLM corpora, prompts, and turn generation procedures

LLM corpus	Prompt	Turn generation procedure
GPT4‐1	Basic prompt	Turn by turn
GPT4‐2	Basic prompt Opening and closing prompt Coordination marker prompt	
Claude‐1	Basic prompt Opening and closing prompt Variation prompt	Turn by turn
Claude‐2	Basic prompt Opening and closing prompt Coordination marker prompt	Turn by turn
Vicuna	Basic prompt Opening and closing prompt Coordination marker prompt	All at once
Wayfarer	Basic prompt Opening and closing prompt Coordination marker prompt	All at once

We instructed LLMs to generate turns in two different ways, either turn by turn, or all at once. In the turn‐by‐turn generation procedure, LLMs were tasked with generating a single turn upon each query, and queries were implemented iteratively within each conversation (see below). In the all‐at‐once generation procedure, LLMs were instructed to produce a complete conversation in each response.

In Study 2, we adopted a complementary, subjective strategy and assessed the ability of human judges to correctly identify randomly sampled short conversational snippets and long excerpts from openings, closings, and topical talk in the main body of human and LLM conversations from Study 1 as either human‐generated or LLM‐generated. The ability of artificial intelligence to pass as human in social interactions with humans relates to the Turing test, variations of which have been extensively discussed from the perspective of various disciplines (Jones & Bergen, 2023, 2024; Pinar et al., 2000). The Turing test does not constitute as straightforward a measure of humanness as is often assumed, especially in a narrow sense based on the intelligence of the interlocutor. However, even if LLM and human spoken conversations differ in their granular features (Study 1), they may still be judged as similar or even indistinguishable by humans, thus constituting a holistic test of the ability of LLMs to simulate human conversation. Many Turing test studies of LLMs exist, but are ambiguous about their ability to deceive humans (Mei et al., 2024). Some suggest that judgments of humanness may be based on stylistic or socioemotional aspects of conversations (Jones & Bergen, 2023, 2024) rather than the highly granular syntactic or lexical features analyzed in Study 1. However, such studies are all based on chat conversations between human judges and LLMs. Adapting the Turing test logic to spoken conversations entails investigating whether LLMs can competently participate in joint social actions (Albert et al., 2024). In Study 2, we thus investigated the ability of human judges to correctly identify whether the spoken conversations generated by LLMs and humans are human or not.

## 2 Study 1

### 2.1 SB conversations

We downloaded SB metadata from https://compprag.christopherpotts.net/swda.html. We initially randomly sampled 200 SB conversations. These conversations, from which transcriber comments were removed, constitute our SB corpus for benchmarking alignment, the use of coordination markers (*oh*, *uh‐huh*, *okay*), and features of openings and closings. The transcripts of many randomly sampled SB conversations did not have an opening or closing. We thus replaced those conversations by randomly sampling the rest of the corpus until reaching a sample of 200 SB conversations for closings. Openings that were transcribed turned out to be very rare. We had to search transcripts for features of openings (like *hello*) and finally obtained a sample of 52 SB conversations for complete openings.

### 2.2 LLM conversations

We compared SB to 200 conversations generated by each LLM using the prompt combinations and turn‐generation procedures in Table 1.

#### 2.2.1 Prompts

The basic prompt imitated the instructions provided to SB participants. It specified the gender, age, and education of participants, as well as the topic (which was matched to the SB conversations). The prompt included instructions to act like a person (with gender, age, and education matched with a corresponding SB conversation) in a phone conversation with someone they did not know. We generated 200 conversations matched with SB conversations for each LLM. Basic prompt instructions also included explanations of the structure of the conversational log. The opening and closing prompt included instructions to create turns exchanging greetings, expressing pleasure at meeting the conversation partners, and asking them how they are doing, where they are calling from, introducing themselves, and other small talk. The coordination marker prompt instructed the LLMs to use *okay*, *oh*, and *uh‐huh* so the conversation is natural. The variation prompt instructed the LLMs to avoid using the same response format over multiple turns.

GPT4‐1 and GPT‐4‐2 conversations were generated using the OpenAI API. Claude‐1 and Claude‐2 conversations were generated with Claude Sonnet 3.5, relying upon the OpenRouter API. Vicuna (lmsys/vicuna‐13b‐v1.5‐16k, downloaded from huggingface.co) conversations were generated on Google Colab on an A100 GPU instance. Wayfarer conversations were generated relying upon the OpenRouter API. The scripts for conversation generation and extension are provided in Google Colab notebook documents on OSF.io (https://osf.io/zxwtr).

#### 2.2.2 Turn generation

The conversations were generated turn by turn for GPT4‐1, GPT4‐2, Claude‐1, and Claude‐2. Turn‐by‐turn generation did not succeed with Vicuna and Wayfarer, which produced multiple turns directly. We suspect Vicuna and Wayfarer failed because of the complexity of the prompt. It is possible that the data used to train and fine‐tune these models did not include similar requests or successful completions. Bowman (2023) noted that smaller LLMs may exhibit random performance for some tasks that larger models successfully complete. In benchmarks, stronger LLMs often outperform weaker ones, even on standard tasks (e.g., LiveBench; White et al., 2024). Even though Vicuna and Wayfarer did not follow the turn‐by‐turn prompt, we decided to go forward, giving them the chance to produce human‐like conversations, as this was the end goal of the task. We thus generated conversational turns for these corpora all at once, that is, each conversation was generated in a single query.

Turn‐by‐turn generation refers to the production of a single conversational turn for each query to the LLM, that is, 50 queries to the LLM return 50 turns of conversation. The prompt for generating each turn included the log of the conversation up to that point. The first turn was generated with “ParticipantA: Hello! <new line> ParticipantB: Hello!”. The roles of the speakers (gender, age, education) were alternated on each query. They were instructed to respond to the last turn of talk but to take the context into account. The prompt included instructions to make the conversation last 50 turns and not initiate the end too early. In testing the prompt, conversations often lasted less than 50 turns, and at times, generation failed. The script contained code for handling exceptions, but in some instances, errors were not caught. In such cases, we resumed turn generation manually.

We thus performed turn generation in successive steps up to a maximum of 50 generated turns per conversation. For the first 20 conversations in the list, we generated 20 turns (this was increased to 30 for the rest of the conversations, as 20 turns was deemed insufficient from inspecting the first 20 conversations). We then extended the conversations that did not have preclosings, to a maximum of 40 turns (adding 10 or 20 turns to the existing conversations). The resulting conversation that did not have preclosings was extended to a maximum of 50 turns (adding 10 turns to these conversations).

LLMs did not always produce the conversations according to the instructions we specified. In GPT4‐1, seven conversations contained multiturn lists of advice and other phenomena that deviated from the expected conversational format requested in the prompt. These conversations were excluded. The number of generated conversations used in analyses on markers and alignment is thus 193. In GPT4‐2, four conversations were excluded from analyses for the same reason. In Claude‐1, two conversations included empty turns, and were excluded. In Claude‐2, one conversation was excluded for this reason. In Vicuna, one conversation was excluded because it included multiturn lists of advice. In eight conversations of this corpus, the LLM repeated the task several times in one response, leading to successive conversations. Only the first such conversation was included. Turns that were manifestly not conversational were also removed (e.g., “Okay, let's begin the conversation”). Also, LLMs did not always generate closings. By the end of the generation procedure, 136 conversations had closings in GPT4‐1, 196 conversations in GP4‐2, 167 in Claude‐1, 158 in Claude‐2, 191 in Vicuna, and 200 in Wayfarer.  These conversations were used for analyses on openings and closings.

### 2.3 Measures

For an example of how measures were annotated, see the Supplementary Materials, File 1, using an example of an LLM conversation (Table S1).

#### 2.3.1 Conceptual, syntactic, and lexical alignment

We used the Python ALIGN package (align‐0.1.1, Duran et al., 2019) to obtain alignment measures between pairs of successive turns in conversations. ALIGN computes one value for conceptual alignment for each turn pair. In other words, conceptual alignment is measured in a single variable. Lexical alignment and syntactic alignment were initially computed for unigrams to trigrams on both tokens and lemmas. Because syntax involves a combination of words, unigrams are not relevant for syntactic alignment. For this reason, we used only the bigram and trigram measures. Because initial measures were highly correlated (all *r*s > .6) within syntactic and lexical alignment, we aggregated them to create one single lexical alignment measure and one single syntactic alignment measure. We used these aggregated variables as well as the conceptual alignment measure as dependent variables in mixed‐model regressions (see below). Before computing the measures, ALIGN automatically removes turns with less than three words and merges successive turns by the same speakers. We report analyses of conceptual, syntactic, and lexical alignment measured between 10 successive pairs of turns (i.e., turn‐pair 1: turn 1 and turn 2, turn‐pair 2: turn 2 and turn 3, turn‐pair 3: turn 3 and turn 4, etc.) in the main body of the conversation, that is, after the topic was initiated (for additional information about computation of measures, see Duran et al., 2019). Topic initiation was annotated as the utterance marking the transition between opening and topic, for example, *okay um well*, *how do you feel about food and cooking?* (inter‐annotator agreement between the second and third author for 193 SB and LLM conversations was high, Cohen's kappa = .90).

We compared alignment between Earlier (average alignment scores for turn‐pairs 1–5) and Later (average alignment scores for turn‐pairs 6–10) sections to assess whether alignment increased over the course of the conversation. In some cases (six SB conversations, five GPT4‐1 conversations, 55 GPT4‐2 conversations, 37 Vicuna conversations, and one Wayfarer conversation) less than 10 turn‐pairs were available for analysis. In those cases, computations were based on the available turn‐pairs. In three SB conversations, two GPT4‐2 conversations, and one Vicuna conversation, only the Earlier section could be computed for this reason.

For each LLM corpus, we report three mixed‐effects regressions in R (using lme4 with the Bobyqa optimizer) predicting conceptual, syntactic, and lexical alignment (dependent variables, DVs) from the independent variables Corpus (LLM or SB), Section (Earlier and Later), and their interaction term. We used conversation ID as a clustering variable to account for the nested structure of the data (sections within conversations; random intercepts). The formula used was: DV ∼ Corpus * Section + (1 | ConvID). We did not fit random slopes because preliminary analyses revealed that doing so would lead to unidentifiable models. Further, random slopes are not necessary to test the effects we are interested in. The reference categories were LLM (e.g., GPT4‐1) for Corpus and Earlier for Section.

#### 2.3.2 Oh, uh‐huh, and okay

For each corpus (LLMs and SB), we computed the number of occurrences and rate per 100 words of *oh*, *uh‐huh*, and *okay* starting from the point of topic initiation until the end of the main body.

#### 2.3.3 Features of openings

Openings (from the first utterance to the utterance before the topic is first initiated) were annotated for the presence or absence of the following features: (1) greeting expressions (e.g., *hello* or *hi*); (2) *okay* (or *all right*, which has similar functions, Bangerter & Clark, 2003); (3) inquiries about the well‐being of the other party or responses to such an inquiry (e.g., *how are you?*); (4) expressions conveying pleasure at meeting the other party (e.g., *nice to meet you*); (5) inquiries (or responses to them) about where someone is located or where they are calling from (e.g., *where are you calling from?*); (6) introductions (e.g., *I'm Doug*); and (7) small talk (talk about a subject other than the prescribed topic but not related to other opening features). Inter‐annotator agreement was high (Landis & Koch, 1977) for all features (all Cohen's kappas ≥ .82).

#### 2.3.4 Features of closings

Closings (from the last topical utterance to the last utterance) were annotated for the presence or absence of the following features: (1) justifications (i.e., expressions indicating a reason for ending the conversation); (2) *okay* or (*all right*); (3) well‐wishing (e.g., *have a nice day*); (4) expressions of pleasure experienced during the conversation (e.g., *it was nice talking to you*); (5) goodbyes (e.g., *goodbye* or *bye*); (6) statements of continuity (e.g., *see you soon*); and (7) thanks (i.e., expressions of gratitude). Inter‐annotator agreement was substantial or higher (Landis & Koch, 1977) for all features (all Cohen's kappas ≥ .72).

### 2.4 Results

#### 2.4.1 Length of turns and opening, closing, and main body phases

For each section of the conversations, we tested the difference between SB and LLM conversations in the mean number of words per turn using regression models comparing SB with each other corpus. Turn length was different between corpora as indicated by the significance of the regression models (openings: *F*(6, 1232) = 444.6, *p* < .001; main body: *F*(6, 1378) = 2471.38, *p* < .001; closings: *F*(6, 1239) = 971.07, *p* < .001).

Overall, LLM conversational turns are much longer than SB conversations (Table 2). The only exception is GPT4‐1 openings, which have about 1.5 words per turn less than SB openings. The largest differences are between SB and Claude 1, with turns longer by a factor of approximately 6 in the main body, and by a factor of 10 in the closing. These huge differences would be difficult to imagine in human spoken conversations, but strongly resemble written conversations (Umair et al., 2024).

**Table 2:** Descriptive statistics and unstandardized regression coefficients for number of words per turn in opening, main body, and closing

Corpus	Words/turn opening		Words/turn main body		Words/turn closing	
	Mean (SD)	B (SE)	Mean (SD)	B (SE)	Mean (SD)	B (SE)
SB, intercept	4.89 (2.03)	4.89 (0.44)^***^	13.97 (5.83)	13.97 (0.59)^***^	5.46 (2.03)	5.46 (0.63)^***^
GPT4‐1	3.05 (1.56)	−1.84 (0.50)^***^	52.74 (9.10)	38.77 (0.84)^***^	29.07 (8.73)	23.60 (1.00)^***^
GPT4‐2	14.39 (4.07)	9.50 (0.50)^***^	56.39 (10.68)	42.42 (0.84)^***^	37.60 (11.75)	32.14 (0.91)^***^
Claude‐1	15.66 (4.52)	10.77 (0.50)^***^	83.57 (11.14)	69.59 (0.84)^***^	55.64 (14.32)	50.17 (0.94)^***^
Claude‐2	16.13 (4.49)	11.24 (0.50)^***^	78.34 (10.31)	64.36 (0.84)^***^	53.82 (12.85)	48.36 (0.96)^***^
Vicuna	7.73 (1.34)	2.84 (0.50)^***^	16.71 (4.27)	2.74 (0.84)^**^	8.60 (2.81)	3.13 (0.91)^***^
Wayfarer	12.39 (2.05)	7.50 (0.50)^***^	19.06 (3.51)	5.08 (0.84)^***^	12.47 (2.42)	7.01 (0.90)^***^

** *p* < .01;

*** *p* < .001.

Topic initiation occurred on average at 12 turns (*SD* = 7.73) in the SB corpus, at 4.27 turns (*SD* = 0.91) in the GPT4‐1 corpus, at 7.7 turns (*SD* = 1.56) in the GPT4‐2 corpus, at 5.87 (*SD* = 0.92) in the Claude 1 corpus, at 6.46 turn in the Claude 2 corpus, at 10.3 (*SD* = 2.73) in the Vicuna corpus and at 9.82 turns (*SD* = 3.11) in the Wayfarer corpus. The differences between corpora were significant, *F*(6, 1231) = 175.99, *p* < .001.

#### 2.4.2 Alignment

We present the descriptive statistics for alignment variables in Table 3. For the mixed effects models analyses, the confidence intervals, standard errors, and variance components, as well as the marginal and conditional pseudo *R* ^2^ (variance explained by the independent variables, and by the independent variables and nesting variables, respectively; Nakagawa et al., 2017) are presented in Table S2 in Supplementary Materials (File 1) for analyses involving GPT4‐1, Table S3 for analyses involving GPT4‐2, Table S4 for analyses involving Claude‐1, Table S5 for analyses involving Claude‐2, Table S6 for analyses involving Vicuna, and Table S7 for analyses involving Wayfarer.

**Table 3:** Means (standard deviations) for conceptual, syntactic, and lexical alignment by corpus and section (earlier vs. later)

Corpus	GPT4‐1		GPT4‐2		Claude‐1		Claude‐2		Vicuna		Wayfarer		SB	
	Earlier	Later	Earlier	Later	Earlier	Later	Earlier	Later	Earlier	Later	Earlier	Later	Earlier	Later
Conceptual	0.80	0.83	0.78	0.81	0.83	0.85	0.84	0.86	0.57	0.55	0.62	0.61	0.57	0.55
	(0.08)	(0.06)	(0.07)	(0.06)	(0.05)	(0.04)	(0.05)	(0.04)	(0.16)	(0.16)	(0.10)	(0.11)	(0.21)	(0.22)
Syntactic	0.28	0.33	0.30	0.35	0.36	0.40	0.34	0.37	0.14	0.15	0.18	0.17	0.16	0.14
	(0.11)	(0.09)	(0.11)	(0.10)	(0.08)	(0.08)	(0.08)	(0.07)	(0.11)	(0.11)	(0.10)	(0.10)	(0.14)	(0.13)
Lexical	0.08	0.10	0.04	0.04	0.04	0.04	0.05	0.06	0.05	0.04	0.03	0.02	0.03	0.03
	(0.07)	(0.07)	(0.04)	(0.04)	(0.03)	(0.03)	(0.03)	(0.03)	(0.08)	(0.07)	(0.05)	(0.04)	(0.06)	(0.06)
*N*	193	193	196	194	198	198	199	199	199	196	200	200	200	197

**Fig. 1:**
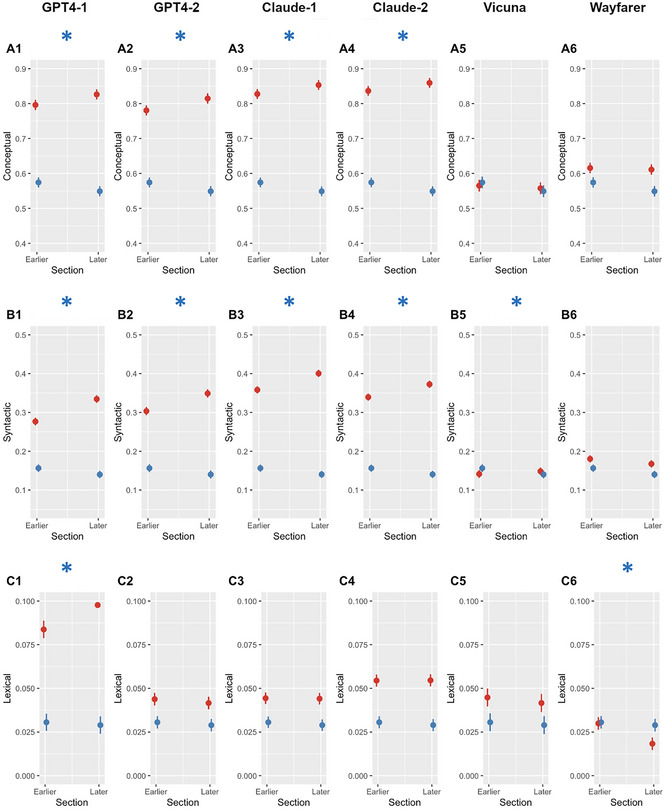
Interactive effects of corpus and section on conceptual, syntactic, and lexical alignment in LLM corpora relative to SB. *Note*. Predicted values are displayed. The SB corpus is indicated in blue and the corresponding LLM corpus is indicated in red. Asterisks indicate that the interaction term is significant in the corresponding model.

Results of the mixed effects model analyses predicting conceptual, syntactic, and lexical alignment by Corpus (SB vs. LLM) and Earlier or Later sections appear in Table 4. The plots of the interactions between Corpus and Section appear in Fig. 1. These are critical analyses because the interaction between Corpus and Section might show that alignment changes between Earlier and Later sections, but only in a specific LLM or in the SB corpus.

**Table 4:** Mixed‐effects model regression coefficients and significance for predicting conceptual, syntactic, and lexical alignment

	Intercept	CorpusSB	SectionLater	Corpus × Section
GPT4‐1				
Conceptual	0.8^***^	−0.22^***^	0.03^**^	−0.06^***^
Syntactic	0.28^***^	−0.12^***^	0.06^***^	−0.07^***^
Lexical	0.08^***^	−0.05^***^	0.01^***^	−0.02^**^
GPT4‐2				
Conceptual	0.78^***^	−0.21^***^	0.03^**^	−0.06^***^
Syntactic	0.3^***^	−0.15^***^	0.05^***^	−0.06^***^
Lexical	0.04^***^	−0.01^***^	0	0
Claude‐1				
Conceptual	0.83^***^	−0.25^***^	0.03^**^	−0.05^***^
Syntactic	0.36^***^	−0.2^***^	0.04^***^	−0.06^***^
Lexical	0.04^***^	−0.01^***^	0	0
Claude‐2				
Conceptual	0.84^***^	−0.26^***^	0.02^*^	−0.05^***^
Syntactic	0.34^***^	−0.18^***^	0.03^***^	−0.05^***^
Lexical	0.05^***^	−0.02^***^	0	0
Vicuna				
Conceptual	0.57^***^	0.01	−0.01	−0.02
Syntactic	0.14^***^	0.01^*^	0.01	−0.02^*^
Lexical	0.04^***^	−0.01^***^	0	0
Wayfarer				
Conceptual	0.62^***^	−0.04^***^	0	−0.02
Syntactic	0.18^***^	−0.02^***^	−0.01	0
Lexical	0.03^***^	0	−0.01^***^	0.01^**^

*Note*. CorpusSB: Estimates for the SB corpus relative to the respective LLM corpus; SectionLater: Estimates for the Later section relative to the Earlier section.

*** *p* < .001;

** *p* < .01;

* *p* < .05.

Conceptual alignment was higher in LLM conversations than SB conversations in the Earlier section, except for Vicuna conversations. It also increased from Earlier to Later sections in the GPT4‐1, GPT4‐2, Claude‐1, and Claude‐2 LLM corpora but not in SB conversations (Fig. 1, Panels A1−A4). Syntactic alignment was higher in LLM than SB conversations in the Earlier section in all comparisons except Vicuna, which had a lower syntactic alignment than SB. It also increased from Earlier to Later sections in LLM conversations involving GPT4‐1, GPT4‐2, Claude‐1, Claude‐2 (Panels B1−B4), but not in SB conversations. Lexical alignment was higher in GPT4‐1 conversations than in SB conversations in the Earlier section. It also increased from Earlier to Later sections in GPT4‐1 conversations (Panel C1), but not in SB conversations. In the GPT4‐2, Claude‐1, Claude‐2, and Vicuna conversations, lexical alignment was also higher than for SB conversations, but did not increase from Earlier to Later sections (Panels C2−C5). In Wayfarer conversations, lexical alignment was not different from SB conversations in the Earlier section, whereas it decreased from the Earlier to Later sections (Panel C6).

Overall, then, GPT4‐1, GPT4‐2, Claude 1, and Claude 2 conversations show greater alignment than SB conversations, and alignment often increases between Earlier and Later sections (albeit less so for lexical alignment). This is in marked contrast to SB conversations. Differences are smaller between Vicuna and Wayfarer and SB conversations. The differences in alignment and their increase may be explained by LLMs’ exaggerated preference for agreement (Supplementary Materials, File 2, Observation 1). For example, GPT4‐1 conversations used *absolutely* as a turn‐initial token of agreement in 24% of all turns. They used *I couldn't agree more* as a token of agreement in an additional 7.5% of turns. Further, LLMs use very uniform syntactic constructions for turn allocation, one example being a tendency to ask questions: All LLM conversations featured significantly more questions than SB conversations (Supplementary Materials, File 2, Observation 7).

#### 2.4.3 Okay, oh, uh‐huh

**Fig. 2:**
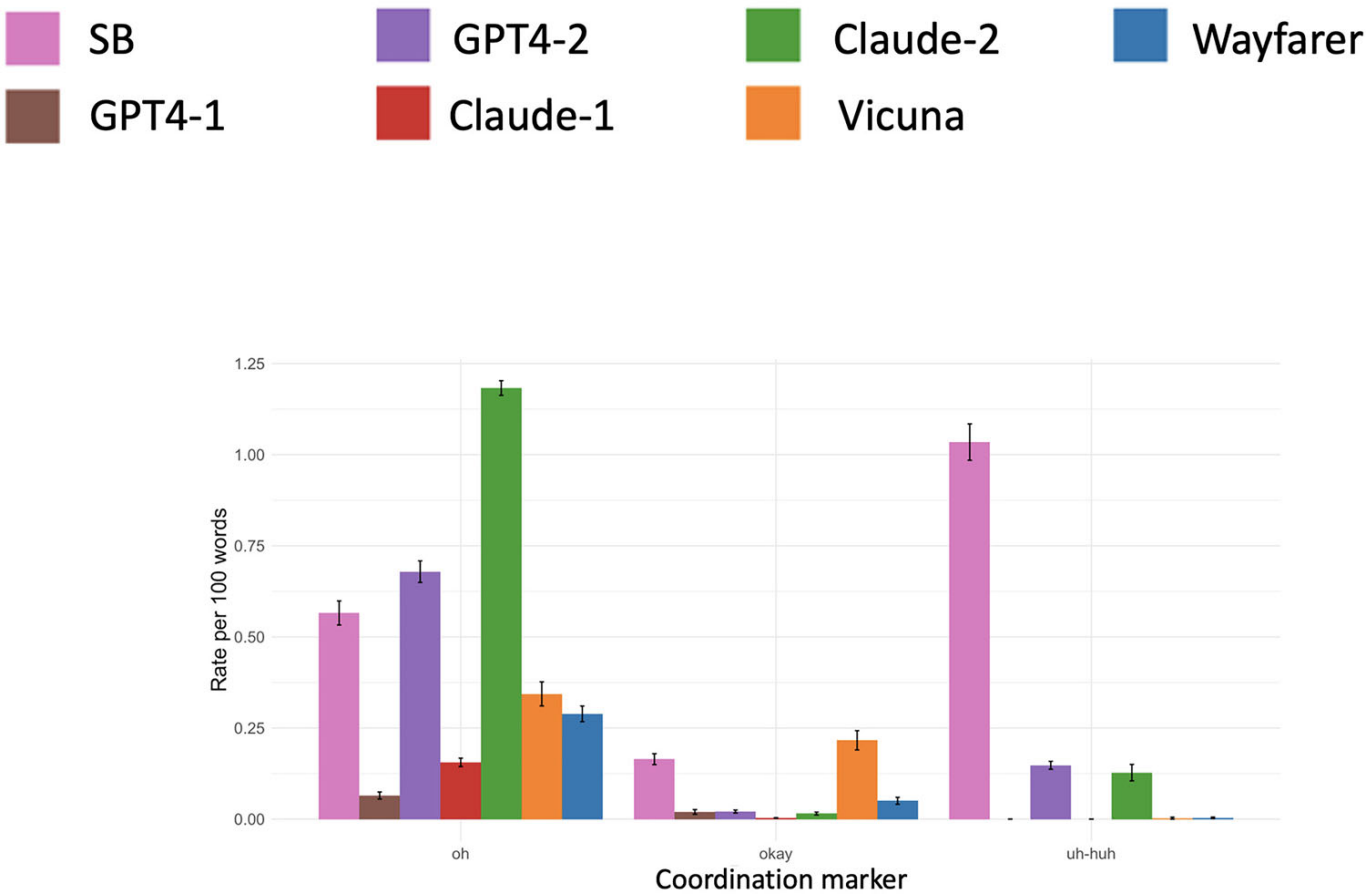
Rates of *oh*, *okay*, and *uh‐huh* per 100 words. *Note*. Error bars represent the standard error of the mean.

We tested the difference between corpora in the rate of *okay*, *oh*, and *uh‐huh* per 100 words using regression models comparing SB with each LLM corpus. The rate of all markers was different between corpora as indicated by the significance of the regression models: *F_oh_* (6, 1378) = 244.09 *p* < .001; *F_uh‐huh_* (6, 1378) = 317.93, *p* < .001; and *F_okay_* (6, 1378) = 45.967, *p* < .001. Table 5 presents the regression estimates for these comparisons between corpora. A visualization of the differences is presented in Fig. 2.

**Table 5:** Regression analysis coefficients comparing SB to LLM corpora in the rate of *oh*, *okay*, and *uh‐huh*

	*Oh*	*Okay*	*Uh‐huh*
	*B* (SE)	*B* (SE)	*B* (SE)
(Intercept)	0.57 (0.02)^***^	0.16 (0.01)^***^	1.03 (0.02)^***^
Claude‐1	−0.50 (0.03)^***^	−0.14 (0.02)^***^	−1.03 (0.03)^***^
Claude‐2	0.11 (0.03)^**^	−0.14 (0.02)^***^	−0.89 (0.03)^***^
GPT4‐1	−0.41 (0.03)^***^	−0.16 (0.02)^***^	−1.03 (0.03)^***^
GPT4‐2	0.62 (0.03)^***^	−0.15 (0.02)^***^	−0.91 (0.03)^***^
Vicuna	−0.22 (0.03)^***^	0.05 (0.02)^**^	−1.03 (0.03)^***^
Wayfarer	−0.28 (0.03)^***^	−0.11 (0.02)^***^	−1.03 (0.03)^***^

*** *p* < .001;

** *p* < .01.

SB conversations featured by far the highest rates of *uh‐huh* per 100 words. This marker was produced very rarely in GPT4‐1, Claude‐1, Vicuna, and Wayfarer conversations. It was produced somewhat more often in GPT4‐2 and Claude‐2 conversations. The rate of *oh* was highest in Claude‐2 and GPT4‐2 conversations, followed by SB, then the other corpora. *Okay* is produced very rarely in all LLM conversations, except for Vicuna, which has a higher rate than SB. Taken together, the production rates of coordination markers in LLM conversations are very different from human conversations.

Not only do LLMs produce coordination markers at different rates, but they also position them in qualitatively different ways. For example, the high rate of *oh* in Claude‐2 conversations is due to the fact that 77% of all turns started with *oh*. Further, when LLM turns featured *uh‐huh*, they often continued by producing a full turn, unlike human conversations where *uh‐huh* is a standalone backchannel (Drummond & Hopper, 1993; Jefferson, 1984). Examples can be found in the Supplementary Materials (File 2, Observation 9).

#### 2.4.4 Openings

**Fig. 3:**
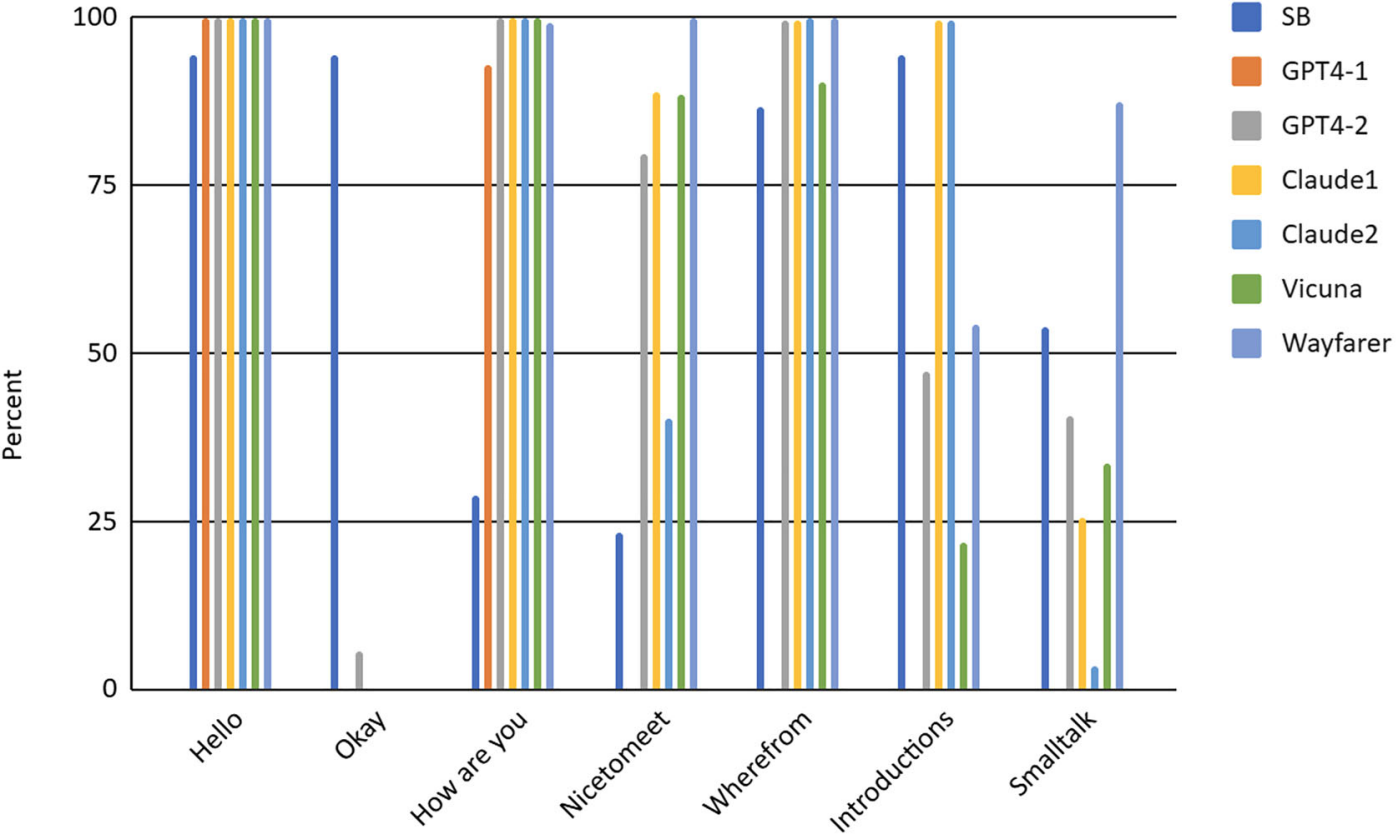
Percentages of *Hello*, *okay*, *How are you*, *Nice to meet you*, *Where are you from*, Introductions and Small Talk in Switchboard and LLM conversation openings.

LLM openings converged with SB in the prevalence of *hello*, all LLMs produced 100% of the time as part of the prompt instructions (compared with 92% of the time in SB openings). LLMs produced *how are you* almost 100% of the time, but this was rather infrequent in SB (<30%). LLMs with enhanced instructions (GPT4‐2 and Claude) produced *where are you from* 100% of the time, approximating SB openings, but GPT4‐1 never did. They also produced *nice to meet you* at >80%, overshooting SB openings (23%). Claude‐1 and Claude‐2 approximated SB well in introductions, but GPT4‐1 and GPT4‐2 did not. LLMs with enhanced instructions approached SB frequencies in small talk. But the biggest difference was in the presence of *okay*, which was used in 94% of SB closings, but almost never in LLM closings, despite enhanced instructions. Taken together, LLMs can reproduce some features of human openings, but not all, and they often show less diversity than human conversations, either producing a feature 100% of the time or almost never (Fig. 3).

#### 2.4.5 Closings

SB and LLM closings converged in expressions of pleasure at the conversation. GPT4‐1 converged with SB for well‐wishing, and GPT4‐2 converged with SB for *goodbye*. For continuity and justification, there was some divergence, whereas okay was highly prevalent in SB closings (83%) but almost completely absent from LLM closings. Taken together, while LLMs do not converge well with SB in closing features, their responses are more diverse than for openings.

Similar to the coordination marker findings, LLM conversations used some features of closings differently from SB conversations. For example, expressions of pleasure were much more effusive (Supplementary Materials, File 2, Observation 10), and expressions of continuity were not just ostensible, but in many cases, led to participants actually making appointments to continue their conversations or to meet later (Supplementary Materials, File 2, Observation 8).

### 2.5 Discussion

Using a combination of LLMs and prompts (Table 1), we investigated whether LLMs are capable of simulating human telephone conversations, using the SB corpus as a reference. Our findings show that LLM conversations differ in several respects from SB conversations. First, conversational turns are much longer in LLM conversations. Second, many LLM conversations feature higher levels of syntactic and conceptual alignment, and moreover, alignment increases over the course of the conversations. Lexical alignment is also higher in many LLM conversations, although its increase is less clear. Third, LLM conversations use coordination markers at different rates and in qualitatively different ways than SB conversations. Fourth, features of openings and closings in LLM conversations are different from SB conversations. In addition to these quantitative findings, we observed several ways in which LLM conversational features differ from typical human conversations (Supplementary Materials, File 2).

**Fig. 4:**
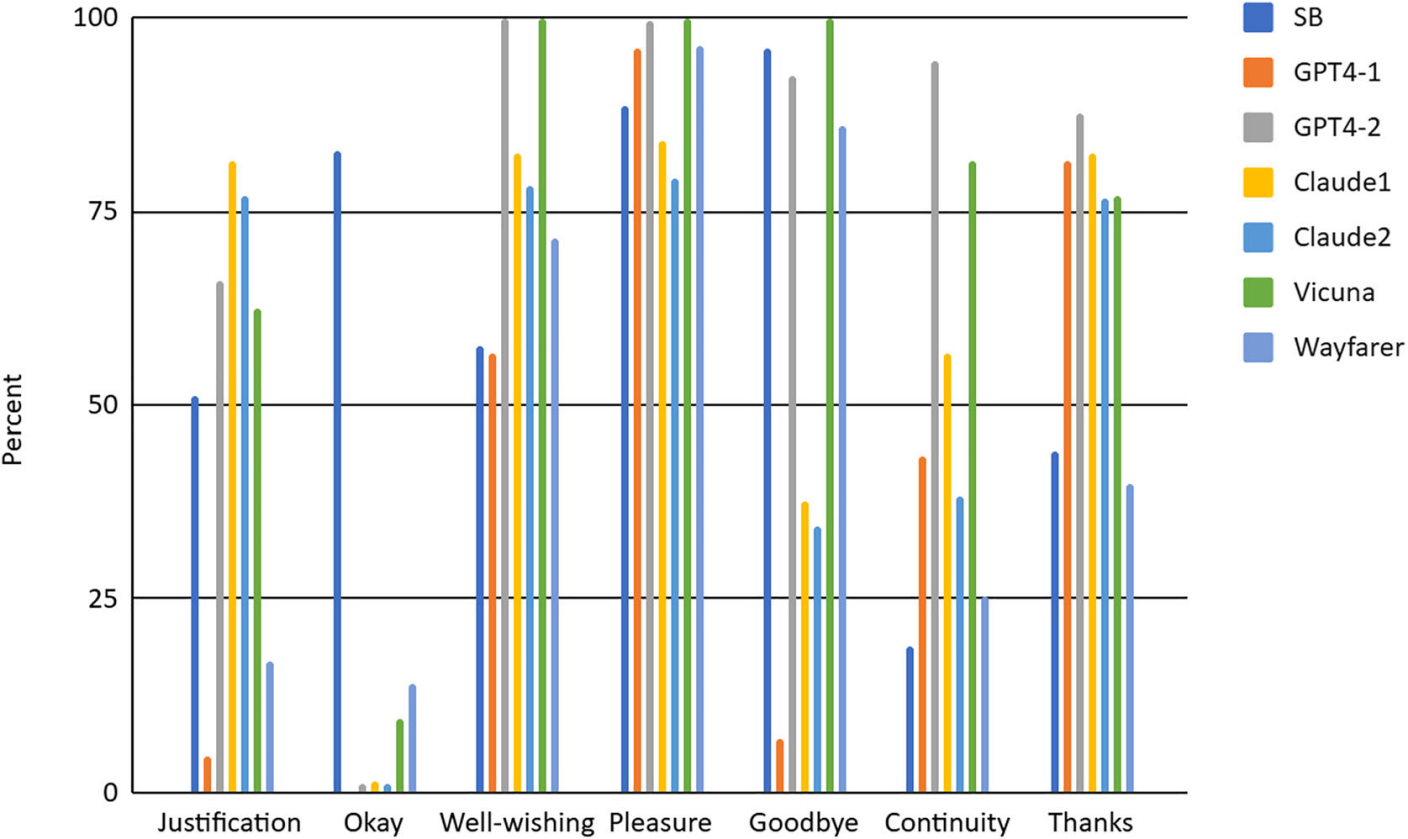
Percentages of Justification, *Okay*, Well‐wishing, Pleasure, *Goodbye*, Continuity, and *Thanks* in Switchboard and LLM conversation closings.

It is noteworthy that Vicuna and Wayfarer conversations more closely resemble SB conversations than the other LLM conversations. This pattern is clearest for alignment (Fig. 1) but also for some other aspects like turn length or the use of *okay* in closings (Fig. 4). This may be due to the construction of the LLMs, but also to the all‐at‐once turn generation instructions used to generate Vicuna and Wayfarer conversations (Table 1).

## 3 Study 2

In Study 2, we tested the ability of human judges to distinguish between SB and LLM conversations. We did this in two ways. First, we constructed short snippets of four turns from the opening phase, main body, and closing phases of SB and LLM conversations and asked participants to judge whether they were human or not. If humanness is immediately evident, SB snippets should be judged as human more often than chance, and LLM conversations less often than chance. Second, we constructed long excerpts of 400 words from SB and LLM conversations and asked participants to judge whether they were human or not. Some of the LLM conversational phenomena documented in Supplementary Materials, such as repetitive turn allocation or systematic use of *oh*, might only become apparent over longer stretches of talk, and thus be more easily detectable from long excerpts.

### 3.1 Participants

We recruited 741 participants from Prolific to judge snippets and 340 to judge long excerpts. The study received ethics approval from the research ethics commission of the University of Neuchâtel. Participants (snippets: mean age 34.4 years, *SD* = 12 years, 54.3% women, 45.2% men, 0.5% undisclosed gender; long excerpts: mean age 37.8 years, *SD* = 13 years, 49.1% women, 50% men, 0.9% undisclosed gender) were screened for English as their primary language. The mean survey completion time was 116.5 s (*SD* = 105 s) for snippets and 283.4 s (*SD* = 192.4 s) for long excerpts.

### 3.2 Materials and methods

For snippets, we randomly selected 10 conversations for each of the opening, main body, and closing of SB conversations (30 conversations in all) and six conversations from each of the opening, main body, and closing of LLM conversations (18 conversations in all for each LLM corpus). We extracted a snippet from each conversation featuring a four‐turn sequence between two speakers transcribed as A and B. Snippets from openings were taken from the first four turns of the conversation. Snippets from the main body were taken from the four turns starting at turn 15 of the conversation. If turn 15 was still in the opening, we counted four turns from the first turn of the main body. Snippets from closings were taken counting back four turns from the last turn of the conversation. Snippets of four turns were chosen to standardize across SB and LLM corpora, but it must be noted that the number of words per turn differs strongly across corpora (Table 1). However, standardizing the number of words would have led to large differences in the number of turns per snippet. Given the interactive nature of spoken conversation, with rapid turn‐taking (Levinson, 2016), we chose to standardize the number of turns rather than the number of words.

Participants were randomly allocated to judge one of the 138 snippets. They first read the following instructions: *You will now see a transcript of the [beginning/middle/end] of a telephone conversation. Please read the transcript and indicate whether you believe it reflects an authentic conversation between two humans or whether it was produced by artificial intelligence* (i.e., *by “bots”*). They were then presented with the snippet and asked to choose whether they thought the conversation was (1) an authentic conversation between humans or (2) produced by bots. Each snippet was judged by between 4 and 6 participants.

For long excerpts, we randomly selected five SB conversations and the corresponding LLM conversations (due to a programming error, only four Claude‐1 conversations were included; we thus had 34 long excerpts in all). Because some conversations were very long, we extracted excerpts of at least 400 words from either the beginning (three conversations) or end (two conversations) of the conversation. That is, we selected excerpts with enough complete turns such that the excerpt featured at least 400 words, but the turn was not truncated. In some cases, this meant the entire conversation was included in the excerpt.

Participants were randomly allocated to judge one of the 34 excerpts (10 participants judged each excerpt). They were incentivized via a one GBP bonus for guessing the correct answer. The instructions read as follows: *You will now see a transcript of part of a telephone conversation. Participants did not know each other and were instructed to talk about a predefined topic. Please read the transcript and indicate whether you believe it reflects an authentic*, *spontaneous telephone conversation between two humans or whether it is a fake conversation produced by artificial intelligence* (i.e., *by “bots”*) *to resemble a conversation between two humans. Please do your best to guess the correct answer. If you guess correctly*, *you will receive a bonus payment of 1 pound*. They then decided whether the conversation was (1) *an authentic conversation between humans* or (2) *produced by bots*.

### 3.3 Results

Results appear in Table 6.

**Table 6:** Judgments of snippets (openings, main body, closing) and long excerpts from SB and LLM conversations as human or LLM‐generated (“bot”)

	Judged “human”	Judged “bot”	Proportion human	*p* binomial
Switchboard				
Opening snippet	14	35	**.29**	.002
Main body snippet	31	20	.61	.080
Closing snippet	22	29	.43	.201
Long excerpt	32	18	.64	.065
GPT4‐1				
Opening snippet	14	16	.47	.708
Main body snippet	8	22	**.27**	.008
Closing snippet	7	22	**.24**	.004
Long excerpt	14	26	.35	.081
GPT4‐2				
Opening snippet	11	18	.38	.132
Main body snippet	7	22	**.24**	.004
Closing snippet	8	22	**.27**	.008
Long excerpt	16	34	**.32**	.015
Claude‐1				
Opening snippet	5	25	**.17**	<.001
Main body snippet	16	13	.55	.356
Closing snippet	11	19	.37	.100
Long excerpt	24	26	.48	.888
Claude‐2				
Opening snippet	12	25	**.32**	.024
Main body snippet	17	18	.49	.500
Closing snippet	23	14	.62	.094
Long excerpt	12	38	**.24**	<.001
Vicuna				
Opening snippet	10	26	**.28**	.006
Main body snippet	22	14	.61	.122
Closing snippet	16	18	.47	.432
Long excerpt	9	41	**.18**	<.001
Wayfarer				
Opening snippet	6	31	**.16**	<.001
Main body snippet	16	20	.44	.309
Closing snippet	18	18	.50	.566
Long excerpt	8	42	**.16**	<.001

*Note*. Proportion “human” judgments significantly different from chance (50%) indicated in bold.

We computed binomial tests to assess whether the proportions were significantly different from chance (Table 6). Participants misjudged SB openings to be produced by bots significantly more than by chance (.29 proportion human), and GPT4‐1 and GPT4‐2 main bodies and closings were significantly more likely than by chance to be judged as produced by bots. Claude‐1, Claude‐2, Vicuna, and Wayfarer openings were significantly more likely than chance to be judged as produced by bots. All other snippets were judged at chance level. Overall, then, GPT4‐1 and GPT4‐2 snippets do not pass for human for the main body and closing phases snippets but pass for opening phases snippets, whereas the reverse is true for Claude‐1, Claude‐2, Vicuna, and Wayfarer. SB long excerpts were correctly identified as human 64% of the time, which did not exceed chance. LLM long excerpts were correctly and significantly identified as produced by bots for GPT4‐2, Claude‐2, Vicuna, and Wayfarer. Claude‐1 and GPT4‐1 long excerpts were judged as produced by humans 48% and bots 35% of the time, respectively, but these proportions were not significantly different from chance.

We also collapsed the snippets across openings, main bodies, and closings, and all LLMs to obtain overall judgments of the humanness of LLM and SB conversations. SB snippets were judged to be human 44% of the time, which is not different from chance (binomial test, *p* = .096), whereas LLM snippets taken together were judged to be human 38% of the time, which is different from chance (binomial test, *p* < .001). By this last result, LLM conversations can be interpreted as unable to convincingly pass as human. This difference in humanness judgments between SB and LLM‐generated snippets is not significant, χ^2^ (1) = 1.75, *p* = .186. We also collapsed long excerpts across all LLMs to obtain overall judgments of the humanness of LLM excerpts. Overall, LLM long excerpts were judged as human 29% of the time (binomial *p* < .001). The difference in humanness judgments between SB and LLM‐generated long excerpts is significant, χ^2^ (1) = 23.85, *p* < .001. Overall, then, there is conclusive evidence that judges are better at discerning between human‐produced and LLM‐produced long excerpts than short snippets.

## 4 General discussion

We investigated the extent to which LLMs can simulate transcripts of spoken human conversations, which differ from chat‐based conversations in the prevalence of features that reflect outputs of real‐time processes of speech comprehension, speech production, and coordination of behavior like turn‐taking or updating common ground or joint commitment.

In Study 1, we compared key features of spoken conversational coordination (conceptual, syntactic, and lexical alignment, openings, closings, and coordination markers like *oh*, *uh‐huh*, and *okay*) in six corpora of LLM conversations using different prompts and turn‐generation procedures and matched to a sample of human telephone conversations from the SB corpus (Godfrey et al., 1992). We found that all forms of alignment were higher in many LLM conversations than in SB conversations, and that alignment increased in LLM conversations between earlier and later sections of the conversations, although this trend was less clear for lexical alignment. Indeed, alignment tended to decrease in human conversations, similar to previous findings (Healey et al., 2014). Thus, alignment patterns are different in human and LLM conversations, although Vicuna and Wayfarer resemble SB more closely than other LLMs. Qualitative observations (Supplementary Materials, File 2) suggest that LLM conversations have less variety than human conversations in both turn construction and turn allocation (Sacks et al., 1974). Turn construction often featured an exaggerated preference for agreement (Lerner, 1996). The repetitive use of these devices thus likely contributed to the high alignment scores of LLM conversations.

Further, we found that LLM conversations differ from human conversations in the prevalence of coordination markers (*oh*, *okay*, *uh‐huh*). The most flexible marker seems to be *oh*, which LLMs were able to produce when responding to the enhanced prompt, albeit not at a similar rate as humans, often overshooting (GPT4‐2 and Claude‐2) or undershooting (GPT4‐1 and Claude‐1) the SB prevalence. Moreover, in LLM conversations, *oh* prefaced the start of long turns, whereas in human conversations, *oh* was also used to preface short turns of persons signaling agreement in a listener role (e.g., *oh for sure*). In LLM conversations, *okay* and *uh‐huh* were very rare, and uses of *okay* were adjectival (e.g., *it's okay to change their mind*, *that's perfectly okay*) rather than as an authentic coordination marker, which was the most frequent use of *okay* in human conversations. Further, *uh‐huh* is often used as a standalone token in human conversations (Drummond & Hopper, 1993; Jefferson, 1984), whereas it often was the first word of a longer turn in LLM conversations.

Finally, the opening and closing phases in human and LLM conversations were different. GPT4‐1 conversation openings were very simple, featuring *hello*s (by instruction) and *how are you*s and nothing else. Human conversational openings reflect in part the real social relationships between participants. Participants in the SB corpus were employees of Texas Instruments, and some discovered that they knew each other, while others were curious about where their conversational partners were calling from. LLMs do not have this social context, and thus, it is not surprising that they did not generate opening features related to it. Enhancing the prompt with explicit instructions to include features of human openings led to an increase in the prevalence of these features in other LLM conversations. But LLM conversations featured less variety than SB conversations. For example, *how are you* and *where are you from* sequences were produced in 99% or 100% of GPT4‐2, Claude‐1, Claude‐2, and Wayfarer conversations. Further, LLM conversations almost never featured *okay*, a common device used to transition from openings to the main body of human conversations (Bangerter et al., 2004).

LLMs sometimes failed to generate closings. When LLM conversations did feature closings, they resembled human closings in some features but were quite different in others, depending on the specific LLM and prompt. For example, LLMs produced highly sycophantic expressions of pleasure at the conversation and exaggerated continuity expressions to the point of agreeing to meet again at a later point in time. Another clear difference is that LLM conversations almost never featured *okay*s, which in human conversations are commonly used to initiate the closing phase (Schegloff & Sacks, 1973).

The features we investigated in Study 1 exhibited limited diversity relative to SB conversations (e.g., the limited range of turn construction and turn allocation procedures, or the all‐or‐nothing nature of the various features of openings, closing, and coordination markers). This corroborates other findings of the lack of diversity in LLM simulations relative to authentic human behavior (Belem et al., 2024; Park et al., 2024). In addition to these phenomena, in the Supplementary Materials (File 2), we documented several other ways in which LLM conversations are qualitatively different from typical human conversations.

In Study 2, we investigated the ability of LLM conversations to pass for human conversations. We presented human judges with short snippets from openings, closings, and main bodies of LLM and SB conversations and long excerpts. For short snippets, we found that all LLMs fail to be perceived as human for parts of the conversations they generated, while passing it for others. For long excerpts, we found that judges could correctly identify LLM conversations at a level much better than chance, suggesting that LLMs clearly fail to be perceived as human. Note that our criterion for passing as human is relatively lenient, because judgment levels that are not different from chance are considered as passing. A stricter criterion according to which judgments should be significantly above the chance level of 50% judgments of humanness to be considered as passing for human was never reached. Thus, while there is substantial uncertainty among judges as to whether snippets are generated by humans or by bots, judges are never confident in misidentifying LLM snippets or long excerpts as being produced by humans. Study 2 findings thus converge with those of Study 1, suggesting that LLMs are not able to produce convincing simulations of human conversations, as judged from a more holistic, subjective perspective. Study 2 findings raise the question of what constitutes a convincingly human conversation, or what “passes” for humanlike conversation. Albert et al. (2024) recently developed a “conversational action test” for assessing the humanness of LLM conversations. If human judges or participants are attentive to the social actions accomplished in (especially spoken) conversations, then LLMs will fail to pass for humans if they are visibly unable to participate competently in social actions.

Our studies have two main limitations. First, although we varied prompts, we cannot be sure that alternative routes of prompt engineering will not yield LLM conversations that better resemble human conversations. A second limitation is that future advances in LLMs (or other currently existing LLMs we have not tested) will be more adept at simulating spoken human conversations, especially if they have been trained with more spoken conversational data. These limitations underscore the lack of a consensus about how to sample LLMs and prompts to ensure the generalizability of claims made about LLM abilities.

Regarding the first limitation, even though we formally investigated the prompts in Table 1, we informally tested several other variants that failed to even generate a single coherent conversation. Further, while the enhanced prompts did lead to the production of additional features (coordination markers, openings, and closings), those were often used very differently than in human conversations. In the Supplementary Materials (File 2), we further documented many other ways in which LLM conversations differ from human conversations. It seems unrealistic to fix all these issues via additional instructions in a prompt.

Regarding the second limitation, it may be difficult for more advanced LLMs to simulate human spoken conversation without being extensively trained on spoken conversation. However, spoken conversational corpora require extensive resources to compile. Thus, the amount of conversational data currently available may not be sufficient to train LLMs on (Dingemanse & Liesenfeld, 2022). And attempts to improve LLM conversational performance by pretraining them on spoken conversations may fail (Umair et al., 2024). Further, some of the current limitations we observed in our LLM‐generated conversations (e.g., exaggerated preference for agreement or enthusiasm) may be caused by issues unrelated to the lack of spoken conversation training, for example, sycophancy, which is a complex and difficult‐to‐solve issue in its own right (Malmqvist, 2024), and whose causes may be independent of a lack of training on human spoken conversation.

To illustrate the difficulty of fixing the current phenomena by training, consider a simple characteristic of LLM conversations, turn length. Turns are 11%−590% longer in LLM conversations than SB conversations. We surmise that many lengthy LLM turns would never be spontaneously spoken out loud by actual humans, because they require cognitive resources in production (e.g., sentence planning) that would excessively tax most human speakers, not to speak of the patience of listeners. The short turns that are the hallmark of human spoken conversations emerge from a real‐time turn‐taking environment where speakers design their turns based on their own cognitive constraints as well as feedback from listeners. Fixing excessively long turns in LLM conversations would require them to first predict potential transition‐relevance places within a longer turn where the other participant might potentially take the floor. Currently, LLMs fail at this task (Umair et al., 2024). They would then need to generate an appropriate response by another participant, also predicting potential floor transitions and keeping track of each participant's own intentions as well as their models of their partners’ intentions. As Dingemanse and Liesenfeld (2022, p. 5616) put it, “Utterances are not just strings with probability distributions defined over them; they stand in relation to other turns, with which they form structured sequences and implement social actions.” A similar case is that of repair, where analyses of human‐LLM chats suggest that LLMs do not have the communicative competence for understanding what it entails (Pütz & Esposito, 2024). Modeling these complex cognitive processes seems difficult, if not impossible, to circumvent using brute‐force approaches, especially given the paucity of spoken conversational data to learn from.

Despite these limitations, our studies contribute to a better understanding of the capacity of LLMs to simulate spoken conversation. Real‐time face‐to‐face spoken conversation features precision timing in the synchronization of multimodal signals exchanged to facilitate the ongoing interpretation of participants’ intentions as the conversation unfolds (Clark, 1996; Holler & Levinson, 2019). This makes spoken conversation fundamentally different from written conversation, and studies of LLM conversational abilities should be mindful of not conflating them. Even spoken conversations via limited media like telephone conversations are multimodal and thus embodied in the material world of visual, audible, postural, and other signals, which go far beyond the purely closed‐circuit linguistic world LLMs inhabit (Barandiaran & Almendros, 2024). We find that LLMs diverge considerably from transcripts of real human telephone conversations, being more strongly aligned, but missing many of the coordination markers of spoken conversations, and potentially suffering from more uniform conversational structure as well as a host of other issues. Thus, it seems unwarranted to assume LLMs will be able to realistically simulate spoken human conversations (Malkiel et al., 2023; Salah et al., 2023) with sufficient granularity in the near future.

To conclude, our studies also underscore the importance of testing LLM conversational performance on their knowledge of spoken conversation as a statistically structured artifact, distinguishable from written conversation by a number of dimensions and constituting a genre of language use distinct from written language (Biber, 1991). This perspective not only allows for more diagnostic evaluation of models but also raises broader theoretical questions about the cognitive mechanisms underlying conversational competence. Returning to Levinson's (2020) interaction engine hypothesis, we also argue that the outputs LLMs generate can serve as probes for the cognitive architecture of interaction. These can be compared to the list of outputs Levinson suggested as typical of the human interaction engine, that is, (1) responding to intentions rather than behaviors, (2) recipient design, (3) indirect coding of intentions in language use, (4) cooperation in making actions interpretable, (5) expectation‐driven sequentiality, (6) role reciprocity, (7) participation structure, (8) close timing, (9) multimodality, and (10) universal properties. That LLMs exhibit some success in mimicking these outputs but fail at many of them implies that only parts of the interaction engine may be emergent from massive exposure to language in context. In many cases, explicit structure or interactional grounding—however they are to be computationally implemented—may still be required to produce competent spoken conversation.

## Data Availability

The data for this project are available on OSF.io: https://osf.io/zxwtr.

## Note

1 We are grateful to the reviewers for suggesting additional LLMs and expanded prompts, which significantly improved the representativeness of our data.
